# Supplementary material for: “Everyone needs a Deb”: what Australian indigenous women say about breast cancer screening and treatment services
Source: BMC Health Serv Res. 2023 Jun 21;23:672. doi: 10.1186/s12913-023-09633-y (PMC10283162; doi:10.1186/s12913-023-09633-y)
Supplement: Supplementary file 1 — Supplementary Material 1 [file 12913_2023_9633_MOESM1_ESM.docx]

**Title:**

“Everyone needs a Deb”: what Australian Indigenous women say about breast cancer screening and treatment services.

Authors:

Vita Christie (1)*, Deb Green (2), John Skinner (1), Lynette Riley(3), Ross O’Shea(4), Karen Littlejohn (4), Christopher Pyke(4), Debbie McCowen(2), Boe Rambaldini (1) and Kylie Gwynne (1)

*Corresponding author: Vita Christie; email: vita.christie@mq.edu.au

Affiliations

1 Djurali Aboriginal and Torres Strait Islander Health Research and Education, Macquarie University; Djurali@mq.edu.au

2 Armajun Aboriginal Health Service, Armidale and Inverell, NSW Australia; dmccowen@armajun.org.au

3 Indigenous Studies & Aboriginal Education, The University of Sydney, NSW Australia; lynette.riley@sydney.edu.au

4 Foundation for Breast Cancer Care, Brisbane Australia; littlejohnkaren67@gmail.com

**Key words: Aboriginal and Torres Strait Islander, Indigenous, breast cancer, culture, screening, perspective, Australia**

**ABSTRACT**

**Background**

Breast cancer continues to be the second most diagnosed cancer overall and the most diagnosed cancer for women in Australia. While mortality rates overall have declined in recent years, Indigenous^[[1]](#footnote-1)^ women continue to be diagnosed at more marginal rates (0.9 times) and are more likely to die (1.2 times). The literature provides a myriad of reasons for this; however, the voices of Indigenous women are largely absent. This study sets out to understand what is happening from the perspectives of Australian Indigenous women with a view to charting culturally safer pathways that improve participation in screening and treatment by Indigenous women.

**Methods**

This co-design study was conducted using semi-structured, in-depth interviews and focus group discussions. Snowball sampling for recruitment of Indigenous women and service providers who were subsequently consented into the study through the Aboriginal Health Service and the research team. Interviews were audio recorded and transcribed verbatim, and data coded in NVivo12 using inductive thematic analysis.

**Results**

A total of 21 Indigenous women and 14 health service providers were interviewed predominantly from the same regional/rural area in NSW, with a small proportion from other states in Australia. Six major themes were identified: Access, Awareness, Community and Family, Lack of control, Negative feelings and associations and Role of services.

**Conclusion**

To improve access and participation of Indigenous women and ultimately improve mortality rates, breast cancer services must explicitly address cultural and community needs.

**INTRODUCTION**

Breast cancer continues to be the second most commonly diagnosed cancer and the most commonly diagnosed cancer for women in Australia (1). While mortality rates overall have declined in recent years, this is not the case for Indigenous women who continue to be diagnosed at more marginal rates (0.9 times) but are more likely to die (1.2 times) than non-Indigenous women (2). For Indigenous women, the five-year relative survival was lowest between the ages of 25–44 and increases thereafter. For non-Indigenous women, the five-year relative survival is similar between the ages of 25-64 and decreases at the ages of 65 or older (2). Overall the five year survival rate was the lowest for Indigenous women; 81% compared to 92% for non-Indigenous women (3). While the disease remains the same, it affects Indigenous women very differently.

The decline in mortality rates across the board is attributed primarily to improved rates of early detection following the introduction of a national population-based mammography screening program (4). Rates have decreased from 74 to 40 per 100,000 women aged 50-74 between 1991- 2014 (5). In contrast, between the years of 1998-2013 Indigenous women did not experience a decline in mortality rates (2). There are several influential factors to explain this. Indigenous women screen at a lower rate than non-Indigenous women (6), are more likely to be younger at diagnosis (7) and the breast cancer is more likely to be further advanced when compared to the non-Indigenous population (2, 3). While age remains the greatest risk factor for breast cancer, Indigenous women are not only younger at the time of diagnosis, they also more likely to receive more invasive surgical treatment compared with non-Indigenous Australians; likely exacerbating the barriers to regular and timely follow-up monitoring and care (8).

The need for improved screening, diagnostic and care pathways for Indigenous women in Australia is well established in the literature (9, 10, 11, 12). The research over the past 20 plus years recognises a variety of barriers and enablers in accordance with more effective breast cancer screening and care for Indigenous women (12, 13, 14, 15, 16, 17, 18, 19, 20). Overwhelmingly, the barriers correlate with cultural safety (12, 14, 15, 16, 19, 20). Health services and programs that are focused on a community led and culturally safe approach also have increased participation rates of Indigenous women in Australia (12, 14, 15, 16). In particular, the integrated involvement of the local Aboriginal community controlled health organisation has shown an increase in screening rates (20). As increased screening rates have been proven to be the most effective way of lowering mortality rates, it is at this stage that efforts should be most concentrated, with the acknowledgement that much needs to change within diagnosis, treatment and follow up services contemporaneously for Indigenous women who require further assessment and treatment.

The voices of Indigenous women has been largely absent in the literature (10). While there has been research into the breast cancer care system and why the statistics are not changing, the authors identified only two other studies about the perspectives of Indigenous women regarding breast cancer screening and treatment in Australia (15, 19). In order to address the gap in breast cancer outcomes, it is very important to deepen our understanding about the experiences of Indigenous women, and to enable culturally safe and timely screening and improvement of the diagnostic and treatment pathways. This study may also provide guidance for improving outcomes for Indigenous women in other high-income countries.

**METHODS**

**Patient and Public Involvement**

Indigenous community organisations, health service providers and members were involved in all stages of the research: conception, design, implementation, evaluation, writing and dissemination.

**Design**

This co-design study adhered to Australia’s National Health and Medical Research Council’s Ethical Guidelines for research with Aboriginal and Torres Strait Islander people (21), a strengths-based approach whereby the Indigenous research team members advice and opinions were privileged.

Semi-structured, in-depth interviews and focus group discussions were conducted over a 1.5 year period (from March 2021- October 2022). Some interviews were via phone (n= 13) (due to Covid restrictions) and the rest were conducted in person (n=22). The interviews were semi-structured based on questionnaires that had been approved by the Aboriginal Health and Medical Research Council.

**Target population and recruitment**

The target population for this study was Indigenous women recruited via snowball sampling from the Aboriginal Health Service (AHS) and other local service providers in a regional/rural area of the Hunter New England region of NSW, Australia. According to ARIA geographical classification scales, the locations are defined as accessible-moderately accessible, having a direct bearing on how many times the BreastScreen mobile van will visit the area (and for how long) and what service delivery takes place onsite, versus the need to travel for services. For example, the largest “accessible” town within the catchment area of this research has an oncology unit but no radiology unit. The area is situated in the Central North of New South Wales and 7.8 percent of the local population identify as Aboriginal and/or Torres Strait Islander (ABS 2021 census data) which is more than double the national population proportion of Indigenous people (3.2%). The five study sites were selected based on pre-existing relationship with the AHS which services the region. The communities expressed interest in working with the AHS in this capacity. The study included participants who were Indigenous women >18 years of age, and other local service providers working with Indigenous women.

All participants were invited to participate by one of the Indigenous co-investigators (DG, JP, GS) who, along with the lead investigator, obtained informed consent via Participant Information Statements and consent forms. The interviewer was not known to the participants prior to the study and the participants were assured their contributions would be deidentified. The majority of service providers were recruited from this area/AHS, and additionally via the McGrath Foundation, which provides Breast Cancer Nurses across Australia. One author (VC) approached the McGrath Foundation with a request for input and was offered an online focus group with nurses working in areas of high Indigenous population from around Australia. All participants were offered a voucher (valued at AUD$50 or AUD$25) as honorarium for their time in participating on the study.

**Data collection and analysis**

The interviews and focus group discussions were audio-recorded, transcribed verbatim and uploaded into NVivo 12. The data were then inductively analysed and coded into overarching themes by one author (VC). Two investigators (VC and KG) discussed each of the themes and sub-themes until they reached consensus, and a third Indigenous investigator (DG) verified the themes. The Summary of themes table (Table 1) was provided to community members to verify it. An Indigenous investigator (DG) facilitated the verification process, going through Summary of themes and subsequently filling in a short survey with the community members. In total, fifteen participants responded in agreement with the themes.

**RESULTS**

A total of twenty-one Indigenous women (of whom, two were survivors) and nine health service providers (seven of whom identified as Indigenous) were interviewed from the same regional area, plus an additional five service providers from various locations in Australia, including remote Western Australia and far north Queensland. Data was collected separately from the women and the service providers but on analysis, the same themes emerged. It was for this reason that the authors decided to report as one group.

The data revealed six key themes: Access; Awareness; Community and family; Lack of control; Negative feelings and associations; and the Role of services and service provision. The themes and key concepts are summarised in Table 1 with accompanying quotes from the community members and the service providers.

**Table 1. Summary of themes emerging from the data**

| **Theme** | **Sub-themes** | **Quotes related to themes** |
| --- | --- | --- |
| 1. **Access -** Enablers and barriers to   accessing services | ENABLERS:   - Cultural safety - Changes in the health system, making it easier - Doing things in the Indigenous way - Culturally appropriate promotional material - Incentive - Support   BARRIERS:   - Hard to access- lacking in resources - Lack of access- distance - Financial barriers to travel - Different care provided according to status (well-resourced people are prioritised)Type of care - painful? invasive? - Opportunistic as opposed to intentional seeking of help or care | *“if women are not aware of what is happening, or how the procedures go, just going in groups makes them feel more confident and more at ease, and then they can talk about their experience when they're finished or before they start. When you see other Aboriginal women it just makes you feel more at ease, or comfortable, about the procedure happening.”*  *“The other thing that struck me when I remember the conversation was that the support services, Aboriginal women would be reluctant to use those services for lack of a better word that they’re run by white women traditionally and I guess it’s about that cultural sensitivity and how those things are handled”*  *“When you see other Aboriginal women it just makes you feel more at ease, or comfortable, about the procedure happening.”* |
| 1. **Awareness**- not only of breast cancer and its determinants, but also of the process of getting screened and where to go for what and what happens when you are there. | - Access to information - Familiarity with disease - Awareness of breast cancer - Understanding breast cancer - Awareness of services | *“…. before (information regarding breast cancer) wasn't out there much. I think we need to have more information – not information, but awareness. Calling groups in, Aboriginal women in, …, to talk about it.”*  *“Whether, it’s the lack of knowledge, or whether it’s taboo, whether it’s a cultural thing. I suppose – yeah, it’s a myriad of variations of why it’s not discussed as openly as such, and I suppose, it’s also dependent on the client, and whether they’re – sort of, don’t want to know that sort of stuff at all. You know? It’s that denial process, as well”* |
| 1. **Community and family**- impact on the family and community and also power of family and community to support people through. | - Impact on family members - Importance of elders and leaders - Learning from others’ experience - Role of family and community - Young women not prioritising health | *“I think if family have an understanding of what’s happening, what to expect, then they’re better equipped to provide that support. I think it’s the barriers. So, again if family know, I know that you’ve got women that just don’t talk about things because, (a) if I talk about it that’s going to put pressure on my family from a financial perspective and, (b) who is going to look after all these children while this is happening because the family isn’t stable? I know that we have a huge amount of people that put their health aside and choose not to address it or pretend it’s not happening, and they’re just a couple of factors. If a family has the knowledge, if family and friends have the knowledge then with family and friends the conversation can be different. “Hey, hang on a minute. This is important. We’re here to support you.” ”* |
| 1. **Feelings/negative associations**- the disease and the experience in the health system of detecting the disease are all unpleasant and scary. | - Anger - Frustration - Fear - Avoiding the topic - Denial - Fear of cancer - Fear of process - Delays in seeking help - Lack of trust - Intention to refuse treatment - Regret - Shame - Trauma | *“And, no matter what cancer someone says they’ve got…..we’re all going to, straight away, think the worst.”*  *“And, I don’t think I’ll go back, either, ‘cause of that experience with the first one”*  *“being quite scared, fearful of knowing. I find that not just with breast cancer, with a lot of conditions. People will choose to just ignore and if they don’t know about it that’s okay.”*  *“Because, the older women are very reserved. They don’t like talking about private areas to the younger generation. They would rather be ridiculed about than talk about it to the younger ones. My elders didn’t talk about it to me.”* |
| 1. **Lack of control -** within the healthcare system and with diseases such as cancer | - At the mercy of the system - Defeatism in face of cancer - Other underlying conditions - Ownership and control of health - Personal choice - Preventable suffering - Status of Indigenous health - Treatment of Aboriginal people and colonisation - Unhelpful advice - Lack of trust in mainstream - No time to prioritise own health | *“Another letter, do they even read it? Just BreastScreen, I'll just chuck it out.”*  *“The other thing I think because it’s predominantly a woman’s issue and mums generally are too busy to have to deal with that and particularly in Aboriginal communities. They have large extended families and a lot of our Aboriginal women are matriarchs in their families as well. Again that comes down to if I don’t know about it I don’t have to deal with it.”* |
| 1. **Role of services** - within community; who does what and who *should* do what | - Role of Aboriginal Health Service - Role of other health services - Role of staff within health services | *“Aboriginal health workers, they’re the ones that’s getting educated around what’s the new package that health is rolling out”*  *“Just (the local Aboriginal health service). We have medical centres and the university here, but I’ve never known anybody else to approach me like that and make themselves be known and this is something that is serious.”*  *“Well we don’t get to talk about that sort of thing in our community because no one comes around and wants to have a talk about it, only when (the local Aboriginal health worker) and them comes up you’re going to. I mean, not in our community, they don’t get together and start to talk about it…. (the local Aboriginal health worker would).. ring us, and “You know, we’re having this, and that….She was so diligent.”* |

**FURTHER FINDINGS AND DISCUSSION**

The themes identified generally aligned with the pre-existing literature (12, 14, 20), and more specifically with the limited literature regarding the perceptions of Indigenous women (15, 19). Overwhelmingly, the data showed that the presence of other Aboriginal people, whether it be as peers, community, support workers, family, health professionals have the biggest influence - *“And, they see that black face... They’re – they’re really pleased. And, you know, they can get the help and support they need.”* One community member specified the role of the Aboriginal Health Workers: *“(They) are going to get you in the door... They’re the ones that’s going to be there to support the client, the culture side of it, but also break down those, we’ve got a different tongue when we talk about health. You’ve got to talk the medical terminology, where we’ve got to have Aboriginal health workers. We’ve got to break that down for our mob.”* One non-Indigenous service provider said *“(The AHW) teaches me how to do it better… as in I’m guided by their beliefs…I find that I learn from them and by involving them, I’m more acceptable to that patient as well”.*

The first theme, Access, covered enablers and barriers from the perspectives of Indigenous women and contained both physical and attitudinal aspects. Whilst some issues of transport are more straightforward to overcome - it is common practice amongst ACCHS to have a fleet of vehicles to provide transport to those in need (22)- in more remote locations, it can be exacerbated by distance and the association of leaving country, with one service provider stating “*when (the women) come to town…. we’ve taken them from country…They’re feeling lousy… but they come to town knowing they need some treatment,…we make them sicker”*. A more obstructive barrier might be the lack of cultural safety as it is complex and requires a multi-faceted approach to overcome. As one community member put it *“if Aboriginal people do not feel safe in a service or feel that it’s culturally respectful and sensitive, they won’t use it.”* Cultural safety can be experienced via physical surrounds, Indigenous support and staffing, being in a group of Indigenous women only or being treated by someone with whom you have developed trust over a period of time. There is a growing body of literature about the ways health services can build and sustain cultural safety including through explicit signs of welcome like Aboriginal and Torres Strait Islander flags in the foyer; zero tolerance of racism; cultural safety training for all staff; employment of Indigenous staff at all levels of the organisation; and engagement with local Elders for advice and trust building (10, 20). One non-Indigenous service provider talked of “the most enormous, beautiful Indigenous artwork about women coming together, supporting each other. It’s really big and it’s the only artwork in the foyer of the breast screen service, but... people all comment on it. It’s more than a painting on the wall”. These concrete strategies could be adopted by breast cancer services and would likely improve access for Indigenous women.

The concept of Awareness emerged very strongly from the data and covered knowledge of services available, knowledge of what will take place in those services and how it might feel, knowledge of breast cancer and how it affects you, and knowledge of how different treatments might be played out. Many of the women felt that holding information/awareness sessions within the AHS would be the easiest and most effective way to deal with this lack of knowledge, as it is a location that the majority felt comfortable with. There was also discussion of what it is that leads to this lack of awareness, encompassing traditional attitudes towards disease and a lack accessible or understandable information; as one woman said *“No one understands doctor jargon”.* This aligns with the pre-existing literature (12).

Community and family are at the centre of Indigenous culture. As one woman put it: *“99 per cent of this community is related by blood… And, regardless if they're related to us or not, if they need help and support, we give it to them.”* While all women agreed that family and community were predominantly the greatest support, it was also clear that the women had differing experiences within their own families and that *“family’s always important, first and foremost, over our own health.”* So, while family is the backbone, it also plays the role of distracting from disease and caring for one’s family can take priority over caring for oneself. It was clear that the more traditional aspect of the culture and the reticence to discuss sensitive health issues both engenders and exacerbates the younger generation feeling powerless in the face of disease- *“I feel like a lot of that too… comes with a lot of the older generations. Like, a lot of the older women, they didn’t really talk about this stuff. They also didn’t have access to, like medical help and all those things. And, so there wasn’t much that they could do to pass on much knowledge about what their daughters should do. Or, what their daughters’ daughters should do. So, it’s just like – I think that’s also a learned behaviour as well.”*. By the same token, having a supportive family and community was central to a better outcome. One non-Indigenous service provider said family *“are almost like the interpreter… in my consults… And, that’s also my link…when we’re trying to get them to town etc,...that other person that came with them, who is some sort of relative to them, is the one that’s often the key person to success”.* This provides important insights about engagement of not only prospective patients, but also the wider community in health literacy about breast cancer and the importance of screening and timely treatment. There were several references to local Elders and the potential impact they had in encouraging women to attend screening and supporting women in breast cancer care, with specific mention of one late Elder *“Aunty (name of elder) … (who) always used to encourage us to go and have a breast screen – in that van. And, like, all the time, you know, she had (breast cancer) too, and … she was sharing some stuff … about it … and she kept encouraging me to go and all that. And, she said, “It’s going to be all right, Sister ...”, “You know, she was there.”*. This expression of gratitude and respect for Elders indicates the strength of their leadership within the community. There was a clear sense from the women that they were part of a bigger picture (the community), and it was suggested that without this connection, they would feel unmoored. Engagement with local Elders is an important strategy for service providers because they provide assurance for other Indigenous women about the safety of services.

The feeling and associations with breast cancer and ill health more generally were overwhelmingly negative. While part of this is due to the traditional attitudes mentioned in the previous paragraph, it is also attributed to fear, shame, embarrassment, anger, frustration and trauma. One service provider explained that clients are *“not used to.. western type medicine.., so they feel a bit late to be in the system in the first place”* and a community member clarified that *“(women are) quite scared, fearful of knowing… not just with breast cancer, with a lot of conditions. People will choose to just ignore and if they don’t know about it that’s okay”.* Another service provider emphasised past experiences as an obstacle, *“the older (women)… know…if you’ve got cancer, you don't come back because they remember that’s what happened to an aunty or another family member”*.

This becomes even more acute as breast cancer affects a private part of the body and is a condition that falls under “women’s business”. In fact, one non-Indigenous service provider mentioned that their local Aboriginal Liaison Officer was male *“so we do find that some of our ladies don’t want to engage because it’s (the) breast”.* There is a feeling that breast cancer healthcare is intimidating; there are no assurances of who the specialist will be or what travel might be required or what to expect when there. The historic and ongoing experiences of Indigenous people with services (*“don’t call it, like, Services NSW because every black fellow’s going to straight away think, Centrelink”* was one community member’s comment) such as healthcare weighs heavily on Indigenous people today and services have a responsibility to explicitly address the barriers.

The lack of control appears to be linked with concepts of self-determination. Multiple women referred to having control over one’s health journey being imperative to improving the status of Indigenous health both within breast cancer and more widely in disease. For example, *“if (white people) want to make decisions about our mob- - - through Closing the Gap. Come on. Get out into the communities and at the grass roots with people… ‘Cause, you don’t know what’s best for our mob. Only us mob know what’s best for our mob.”*

While there was much discussion about the challenges of the current healthcare system, there was conversely a clear sense of the potential role of services within community. Mainstream health services were cited as having *“resources, but they can’t get out into the community because it doesn’t allow them to do that”*. While the women spoke of a lack of services or ineffectual health promotion -*“for a town as big as this, I’m very, very alarmed that the lack of support services”*- they also spoke in glowing terms of their experience with the local AHS and their gratitude to the Indigenous leaders within the community. In fact, it was a common suggestion that the screening take place on the AHS premises, allowing for opportunistic and culturally welcoming service delivery. The women recognised the imperative role of Aboriginal Health Workers to locally coordinate and with the various health care providers, including Breast Screen, doctors and specialists and the women agreed *“Everyone needs a Deb”*. This referred to the local Aboriginal Health Worker who corresponded with BreastScreen, contacted the women directly via phone to encourage them to attend screening, contacted women who were suffering to see if they needed anything, organised vehicles to pick people up, stopped people in the supermarket to encourage them to attend screening, organised information sessions for the women and assisted women through the system to ensure feelings of comfort and control. The success of any programs to improve breast cancer care for Indigenous women appear heavily reliant on this ‘champion’ and there has recently been extensive discussion of the role of Indigenous patient navigator in countries other than Australia (23, 24, 25), with the recommendation to promote the role within Australia given its success.

**CONCLUSION**

Knowing the major concerns and what are considered the greatest barriers and enablers for Indigenous women to participate in breast cancer diagnosis and treatment services gives us clear direction as to how Australia’s approach must change. Engaging Indigenous women in breast cancer diagnosis and treatment cannot remain simply an intention, it will require a new approach. Indigenous women are saying that culture, accessible information, and Indigenous ways of knowing, being and doing are the key to increasing engagement and enabling access.

While this information is not new (15, 19), the continuing lack of improvement in outcomes for Indigenous women is evidence that the information has not yet been effectively incorporated into policy or practice.

The data clearly indicates that there are multiple ways in which services could be improved to better suit the needs of Indigenous women. We learn this directly from the perspectives of Australian Indigenous women and service providers in breast cancer care. We can apply this generously shared knowledge to effect change, remembering that if we want to reduce mortality and achieve parity for Indigenous women, services must explicitly address communities’ cultural needs.

**Declarations**

**Ethics approval and consent to participate**

This study has ethics approval from Aboriginal Health and Medical Research Council (AH&MRC), Ref: 1525/19. All methods were performed in accordance with the relevant guidelines and regulations.

All persons who provided data for this study signed an informed consent.

**Consent for publication**

Not applicable

**Availability of data materials**

The data collected and analysed during this study are available from the corresponding author upon reasonable request.

**Competing interests**

None declared

**Funding**

This research received no specific grant from any funding agency in the public, commercial or not-for-profit sectors

**Authors contributions**

Conception and design of the study - KG, DG, VC

Planning- VC, KG, DG

Governance and scientific advice – KG, CP, DM, DG, VC

Conduct and reporting: VC, KG, DG

Drafting the Manuscript – VC, KG

Review and approval of the manuscript – all authors

**Acknowledgements**

We would like to acknowledge the time and assistance of the staff of Armajun Aboriginal Health Service, in particular Jody Pitt and Gina Shepherd.

**REFERENCES**

1. Cancer Australia. Breast Cancer 2022 [Statistics for Breast cancer]. Available from: <https://www.canceraustralia.gov.au/cancer-types/breast-cancer/statistics>.

2. Australian Institute of Health and Welfare. Cancer in Aboriginal & Torres Strait Islander people of Australia. Canberra: AIHW; 2018.

3. National Breast Cancer Foundation. Aboriginal and Torres Strait Islander peoples and breast cancer 2022

4. Australian Institute of Health and Welfare. BreastScreen Australia monitoring report 2018. Canberra: AIHW; 2018.

5. Australian Institute of Health and Welfare. BreastScreen Australia monitoring report 2022. Canberra: AIHW; 2022.

6. Moore SP, Soerjomataram I, Green AC, Garvey G, Martin J, Valery PC. Breast cancer diagnosis, patterns of care and burden of disease in Queensland, Australia (1998-2004): does being Indigenous make a difference? Int J Public Health. 2016;61(4):435-42.

7. Dasgupta P, Baade P, Youlden D, Garvey G, Aitken J, Wallington I, et al. Variations in outcomes for Indigenous women with breast cancer in Australia: A systematic review. European Journal of Cancer Care. 2017;26(6):e12662.

8. Banham D, Roder D, Keefe D, Farshid G, Eckert M, Howard N, et al. Disparities in breast screening, stage at diagnosis, cancer treatment and the subsequent risk of cancer death: a retrospective, matched cohort of aboriginal and non-aboriginal women with breast cancer. BMC Health Services Research. 2019;19(1):387.

9. Thompson SC. Indigenous women and breast cancer. Medical Journal of Australia. 2009;190(10):602.

10. Christie V, Green D, Amin J, Pyke C, Littlejohn K, Skinner J, et al. What Is the Evidence Globally for Culturally Safe Strategies to Improve Breast Cancer Outcomes for Indigenous Women in High Income Countries? A Systematic Review. International Journal of Environmental Research and Public Health. 2021;18(11).

11. Christie V, Rice M, Dracakis J, Green D, Amin J, Littlejohn K, et al. Improving breast cancer outcomes for Aboriginal women: a mixed-methods study protocol. BMJ Open. 2022;12(1):e048003.

12. Byers L, Michell K, McCullough K. Awareness, acceptability and access to screening mammography for remote Aboriginal women. Health Promot J Austr. 2018;29(3):366-7.

13. Supramaniam R, Gibberd A, Dillon A, Goldsbury DE, O’Connell DL. Increasing rates of surgical treatment and preventing comorbidities may increase breast cancer survival for Aboriginal women. BMC Cancer. 2014;14(1):163.

14. Hedges S, Davidson M, Forrester S, Casey A, Pridmore V, Cooper A, et al. A Breast Screening Shawl to Help Aboriginal Women Feel More Comfortable and Culturally Safe. Journal of Global Oncology. 2018;4(Supplement 2):40s-s.

15. McMichael C, Kirk M, Manderson L, Hoban E, Potts H. Indigenous women's perceptions of breast cancer diagnosis and treatment in Queensland. Australian and New Zealand Journal of Public Health. 2000;24(5):515-9.

16. Fox W, Powell M, Hyland V, Honeyball F, editors. Supportive care for women with breast cancer living in rural Australia. Cancer Forum; 2017: The Cancer Council Australia.

17. 'Orchid' Breastscreen Queensland's New 4WD Digital Mobile Service. 2010-08;34(5):39.

18. Campbell J, Kurnoth P. Well women making a diffence: evaluation report and program guide. Darwin: Women's Cancer Prevention Program, Department of Health and Community Services, Northern Territory Government; 2000.

19. Pilkington L, Haigh MM, Durey A, Katzenellenbogen JM, Thompson SC. Perspectives of Aboriginal women on participation in mammographic screening: a step towards improving services. BMC public health. 2017;17(1):697.

20. Reath J, Carey M. Breast and cervical cancer in indigenous women-overcoming barriers to early detection. Aust Fam Physician. 2008;37(3):178-82.

21. National Health and Medical Research Council. Ethical conduct in research with Aboriginal and Torres Strait Islander Peoples and communities: Guidelines for researchers and stakeholders. Canberra: Commonwealth of Australia; 2018.

22. The Centre for Research Excellence in Aboriginal Chronic Disease Knowledge Translation and Exchange (CREATE). Aboriginal Community Controlled Health Organisations in practice: Sharing ways of working from the ACCHO sector. online: South Australian Health and Medical Research Institute, Adelaide.; 2020.

23. Rankin A, Baumann A, Downey B, Valaitis R, Montour A, Mandy P. The Role of the Indigenous Patient Navigator: A Scoping Review. Canadian Journal of Nursing Research. 2022;54(2):199-210.

24. Grimes C, Dankovchik J, Cahn M, Warren-Mears V. American Indian and Alaska Native Cancer Patients’ Perceptions of a Culturally Specific Patient Navigator Program. The Journal of Primary Prevention. 2017;38(1):121-35.

25. Hiscock EC, Stutz S, Mashford-Pringle A, Tan S, Scott B, Oblin-Moses L, et al. An environmental scan of Indigenous Patient Navigator programs in Ontario. Healthcare Management Forum. 2022;35(2):99-104.

1. The authors use the word Indigenous when referring to Aboriginal and Torres Strait Islander people from Australia on advice of one of the Indigenous authors. [↑](#footnote-ref-1)
